# Filaricidal activities on *Onchocerca ochengi* and *Loa loa*, toxicity and phytochemical screening of extracts of *Tragia benthami* and *Piper umbellatum*

**DOI:** 10.1186/s12906-016-1319-2

**Published:** 2016-08-30

**Authors:** Fidelis Cho-Ngwa, Elvis Monya, Boris K. Azantsa, Faustin Pascal T. Manfo, Smith B. Babiaka, James A. Mbah, Moses Samje

**Affiliations:** 1 ANDI Centre of Excellence for Onchocerciasis Drug Research, Biotechnology Unit, Faculty of Science, University of Buea, P.O. Box 63, Buea, Cameroon; 2 Department of Chemistry, Faculty of Science, University of Buea, P.O. Box 63, Buea, Cameroon; 3 Department of Biomedical Sciences, Faculty of Health Sciences, University of Bamenda, P.O. Box 39, Bambili, Cameroon

**Keywords:** Onchocerciasis, *Loa loa*, *Tragia benthami*, *Piper umbellatum*

## Abstract

**Background:**

Onchocerciasis is the world’s second leading infectious cause of blindness. Its control is currently hampered by the lack of a macrofilaricidal drug and by severe adverse events observed when the lone recommended microfilaricide, ivermectin is administered to individuals co-infected with *Loa loa*. Therefore, there is the need for a safe and effective macrofilaricidal drug that will be able to cure the infection and break transmission cycles, or at least, an alternative microfilaricide that does not kill *L. loa* microfilariae (mf).

**Methods:**

Fourteen extracts from two medicinal plants, *Tragia benthami* and *Piper umbellatum* were screened in vitro against *Onchocerca ochengi* parasite and *L. loa* mf. Activities of extracts on male worms and microfilariae were assessed by motility reduction, while MTT/Formazan assay was used to assess biochemically the death of female worms. Cytotoxicity and acute toxicity of active extracts were tested on monkey kidney cells and Balb/c mice, respectively.

**Results:**

At 500 μg/mL, all extracts showed 100 % activity on *Onchocerca ochengi* males and microfilariae, while 9 showed 100 % activity on female worms. The methylene chloride extract of *Piper umbellatum* leaves was the most active on adult male and female worms (IC_50_s: 16.63 μg/mL and 35.65 μg/mL, respectively). The three most active extracts on *Onchocerca ochengi* females were also highly active on *Loa loa* microfilariae, with IC_50_s of 35.12 - 13.9 μg/mL. Active extracts were generally more toxic to the worms than to cells and showed no acute toxicity to Balb/c mice. Phytochemical screening revealed the presence of saponins, steroids, tannins and flavanoids in the promising extracts.

**Conclusions:**

These results unfold potential sources of novel anti-*Onchocerca* lead compounds and validate the traditional use of the plants in onchocerciasis treatment.

## Background

Human onchocerciasis also known as river blindness is a neglected tropical disease caused by the filarial worm, *Onchocerca volvulus*. It is the world’s second leading infectious cause of blindness, transmitted by black flies of genus *Simulium* [1]. An estimated 37 million people in tropical countries are infected with the parasite, with approximately 99 % of all cases in Africa [1]. Adult worms of *O. volvulus* live in subcutaneous nodules for more than 14 years and produce millions of microfilariae (mf), which parasitize skin and eye tissues, resulting often in debilitating pathologies, which include blindness [2]. On the other hand, loasis, caused by the filarial worm, *Loa loa,* prevalent mainly in the rain forests of West and Central Africa is not as severe as the other filariasis and is consequently less well studied [3, 4]. *L. loa* and *O. volvulus* are often co-endemic, infecting the same individuals, rendering the control of onchocerciasis somewhat difficult.

Current control measures for onchocerciasis rely heavily on the community directed treatment with the only recommended drug, ivermectin. However, the use of this drug is limited in areas of *L. loa* co-endemicity due to severe adverse events observed in people with high *L. loa* microfilaraemia because of the good activity of the drug on the *L.loa* mf in blood [5]. Moreover, ivermectin is only microfilaricidal and requires continuous delivery for at least 14 years (which corresponds to the life span of the adult worm) to interrupt transmission [5]. Lastly, parasites isolated from communities with sub-optimal responses to annual treatment in Ghana showed genetic changes observed with resistance to ivermectin in other nematodes [6]. Therefore, there is the need for a safe and effective macrofilaricidal drug against onchocerciasis that will be able to cure the infection and break transmission cycles, or at least, an alternative microfilaricide that does not kill *L. loa* mf. Such a drug has been difficult to come by, especially considering for-profit pharmaceutical companies, requiring alternative strategies to aid its discovery and development.

Natural medicines, including medicinal plants, have played a very significant role in the health care of rural populations in Africa and other developing countries [7]. The majority of drugs active against infectious agents are in fact, derived from natural products [8]. For example, ivermectin itself is isolated from *Streptomyces avermitilis* [9], and the malaria drug, artemisinin, derived from the medicinal plant, *Artemisia annua* [10]. Previous studies have revealed the filaricidal effect of some Cameroonian medicinal plants [11–13]. Many of the studies have been based on the bovine derived *Onchocerca ochengi* (*O. ochengi*), currently known to be the closest relative and most suitable model of *Onchocerca volvulu*s, which is difficult and expensive to obtain from humans [14]. The present study was therefore, carried out to assess and potentially exploit the acclaimed anti-filarial properties of *Piper umbellatum* L and *Tragia benthami* B, two plants used in the traditional treatment of onchocerciasis in Cameroon, on *O. ochengi* and *L. loa. T. benthami* is a herbaceous climber or trailer, with stinging hairs, of the family *Euphobiaceae* found in West Africa, mostly Cameroon, along the banks of fast running streams. Studies have shown the antibactericidal, antifungal, antipyretic and analgesic properties of ethyl acetate and ethanol extracts of a related *Tragia* species [15, 16]. *P. umbellatum,* on the other hand, is a tropical shrub of the family *Piperaceae* with many nutritional and medicinal values in different parts of Nigeria. Its extracts have been demonstrated to exhibit contraceptive and bactericidal activities [17–19].

## Methods

### Collection, identification and processing of plants

The plants, *P. umbellatum* (voucher number 19813) and *T. benthami* (voucher number 33628) were harvested in the village of Santa Mbei in the North West Region of Cameroon, based on ethnobotanical and pharmacological information. Voucher specimens were taken to the National Herbarium of Cameroon, Yaoundé, where they were authenticated by a botanist and voucher numbers assigned to them. The leaves and roots of *T. benthami* and the leaves, roots and seeds of *P. umbellatum* were air dried to constant weight and then ground to fine powder.

### Preparation of crude extracts and stock solutions

Each powder was macerated for 48 h, sequentially, three times per solvent, in hexane, methylene chloride, and then methanol. The filtrates were concentrated using a rotary evaporator (BUCHI Rotavapor R-200, Switzerland) at the boiling points of the solvents. Each crude extract was recovered with a small volume of methylene chloride and left on the shelf for 72 h for any residual solvent to evaporate. The dried extracts were then weighed, and the percentage yield calculated. Stock solutions of 25 mg/mL were also prepared from each extract in dimethyl sulfoxide (DMSO, solvent grade >99.8 %, from Sigma-Aldrich, Germany) and kept at −20 °C until tested on worms and larvae.

### Isolation and screens on *Onchocerca ochengi* adult worms

#### Isolation of *O. ochengi* adult worms (macrofilariae)

Worms were isolated by the method described by Cho-Ngwa et al. [20]. Briefly, fresh pieces of umbilical cattle skin with palpable nodules bought from slaughter houses in Douala and Buea, Cameroon, were thoroughly washed with soap and water, and the inner and outer surfaces sterilized with 70 % ethanol which was allowed to evaporate in a lamina flow sterile hood. Nodules were carefully excised, and the recovered worm masses submerged in complete culture medium (CCM, which is RPMI 1640 with NaHCO_3_ and supplemented with 25 mM HEPES, 0.3 g γ-irradiated L-glutamine powder, 5 % newborn calf serum, 200 units/mL penicillin, 200 μg/mL streptomycin and 0.25 μg/mL amphotericin B; pH 7.4) in 24-well culture plates (NUNC, USA). The plates containing the worms were incubated overnight at 37 °C under an atmosphere of 5 % CO_2_ in humidified air (in a HERACELL-150i CO_2_ incubator, USA). Thereafter, the viability of worms and sterility of cultures were evaluated using an inverted microscope (Nikon Eclipse TS100, China) prior to drug testing in primary and secondary screens.

#### Primary screens on *Onchocerca ochengi* adult worms

The primary screen was done in order to eliminate inactive extracts. The worms were treated in quadruplicates with either an extract at 500 μg/ml in CCM, or auranofin at10 μM (serving as positive control) [21] or 2 % DMSO (negative control), and incubated for 5 days at 37 °C in 5 % CO_2_ atmosphere, and their viabilities assessed. The 2 % DMSO was shown to be safe for worms in our previous studies [11–13].

Adult male worm viability was assessed through evaluation of worm motility using an inverted microscope. Activity scores ranged from 100 % (complete inhibition of motility), 75 % (only head or tail of worm shaking occasionally), 50 % (whole worm motile, but sluggishly), 25 % (only little change in motility), to 0 % (no observable change in motility).

Activity of extracts on female worms was assessed biochemically, by visual estimation of percentage inhibition of formazan (blue colour) formation following incubation of the nodules in 500 μl of 0.5 mg/ml MTT (3-(4, 5 dimethylthiazol-2-yl)-2, 5-diphenyltetrazolium bromide, from Sigma) for 30 min [11, 12]. Activity scores assigned ranged from 100 % parasite killing (no blue formazan coloration seen), 90 %, 75 %, 50 %, 25 %, to 0 % (entire worm appears blue as in negative control).

An extract was considered active if there was ≥ 90 % inhibition of male worm motility or of formazan formation; moderately active if there was a 50–89 % inhibition of male worm motility or of formazan formation and inactive if there was a < 50 % inhibition of male worm motility or of formazan formation. All experiments were repeated once prior to the secondary screens.

### Isolation of and screens with *Onchocerca ochengi* microfilariae

#### Preparation of mammalian cells

LLC-MK2 obtained from American Type Culture Collection (ATCC, Virginia, USA) were proliferated in CCM at 37 °C under an atmosphere of 5 % CO_2_ in humified air. The cells were grown in 96-well plates until they became fully confluent, and served as feeder layers for the microfilariae (mf) assays. The cells were also used for cytotoxicity assessment of the extracts [13].

#### Isolation of *Onchocerca ochengi* microfilariae

This was by the method of Cho-Ngwa et al. [11] with slight modifications. Briefly, umbilical cattle skin pieces containing palpable nodules were obtained from the abattoir, cleaned, carefully shaved, and sterilized with 70 % ethanol. Skin slivers were obtained and incubated for 4–6 h at room temperature in CCM and the emerged and highly motile *O. ochengi* mf were concentrated by centrifugation (400×g, 10 min). The mf were re-suspended in CCM and distributed into wells (about 15 mf/100 μL of CCM/well) of the 96-well plates containing the LLC-MK2 cell layer, and their viability and sterility ascertained for 24 h prior to addition of extracts.

#### Primary screens on *O. ochengi* mf

This was done as previously described [11]. Primary screens were done at 500 μg/mL in duplicates, in order to eliminate inactive extracts. The mf were incubated at 37 °C under an atmosphere of 5 % CO_2_ in humidified air for 5 days. Mf motility was scored microscopically, daily. The percentage motility inhibition scores were assigned as 100 % (all mf immotile), 75 % (only head or tail of mf shaking, occasionally), 50 % (whole body of mf motile but sluggishly or with difficulties), 25 % (almost vigorous motility), 0 % (vigorous motility).

#### Secondary screens on *O. ochengi* adult worms and mf

This was done as previously described [11]. Extracts with 100 % activity on both adult female worms and mf at primary screens were re-tested at serial dilutions of eight concentrations (spanning the range of 500 to 3.91 μg/mL), in order to determine the IC_50_ values using Graphpad prism software, version 6.0 (GraphPad Prism INC., CA, USA).

### Isolation and screens on *Loa loa* microfilariae

#### Isolation of *Loa loa* mf

Ethical clearance (2013/11/371/L/CNERSH/SP) and administrative clearance (631–06.14) were obtained from the Cameroon National Ethical Committee and the Ministry of Public health, respectively. Local administrative clearance was given by the District Medical Officer of the Edea health district where patients were recruited for the study. Informed consent was obtained freely from individuals who tested positive for high *L. loa* mf load.

Isolation of *L. loa* mf was done as previously described by Gousard et al. [22] but with some modifications. Freshly collected *L. loa* -infected blood (2 mL) was diluted (1:2) with incomplete culture medium (ICM; i.e., CCM without serum) and carefully layered on 4 ml of Ficoll-pacque (TM) in a 15 mL centrifuge tube. The tube was spun in a swing bucket centrifuge (400×g, 15 min, 25 °C), and the ficoll layer containing mf recovered and washed three times with ICM. The mf were re-suspended in CCM, and then distributed in wells of a 96-well microtiter plate containing LLC-MK2 cell layers (~15 mf/well/100 μL of CCM). The mfs were monitored for 24 h for viability and sterility before treatment with extracts.

#### Screens on *L. loa* mf

Only the most active extracts on *O. ochengi* adult female worms and mf were screened on *L. loa* mf. This was done as in the primary *O. ochengi* mf screen and at serial dilutions of 8 concentrations (500–3.91 μg/mL).

### Toxicity assessment of extracts

#### Cytotoxicity assessment of extracts

Cytotoxicity of extracts with good filaricidal activities were assessed on LLC-MK2 cells on day 5 as part of the mf secondary screen assays as previously described [11]. The 50 % cytotoxic concentrations (CC_50_ values) for these cells were determined by microscopic examination. Dead or deformed cells were usually detached from the bottom of the vessel and were rounded in shape. The selectivity index (SI) of each extract was calculated as the ratio of CC_50_ of the extract on the mammalian cells to the IC_50_ of the extract on the *O. ochengi* or *L. loa* parasite.

#### Acute toxicity

The acute toxicity was evaluated for the most active extracts on *O. ochengi* adult worms and mf in accordance with the Organisation for Economic Co-operation and Development (OECD) guidelines for testing chemicals [23]. Ethical permit was obtained from the University of Buea, Faculty of Science Institutional Animal Care and Use Committee (IACUC). Eight nulliparous and non-pregnant Balb/c mice, ten weeks old (averagely 18 g each) were divided in four groups (2 per group) and kept in their cages for 5 days to allow for acclimatization to the new housing conditions. Three groups were used for the three most active extracts and the last group as negative control. Food, but not water was withheld for 4 h after which the animals were weighed and the extract administered orally using a gavage needle at a limit dose of 2000 mg/kg body weight with a volume of 1 mL/100 g body weight of mice. The extracts were diluted with distilled water prior to administration, while negative control mice received the vehicle (2 % DMSO diluted in distilled water, 200 μL/kg body weight). After administration of the extracts, food was withheld further for 2 h. The animals were observed individually after dosing, once every 30 min during the first 4 h. The animals were weighed every 2 days and observed for physical activity and behavioural pattern, food and water intake, changes in skin and fur, eyes and mucous membranes, tremours, convulsions, salivation, lethargy, sleep, coma and death for 14 days.

#### Phytochemical screening

Phytochemical derivatives in the 3 most active extracts were investigated by standard methods against known references. The presence of saponins and sterols was determined as described earlier [24]. Saponins were detected by dissolving trace amounts of the extract in distilled water and shaking thoroughly. Frothing which persisted on warming was observed and taken as evidence. The presence of steroids was determined by dissolving 0.1 g extract in methylene chloride (3 mL) and acetic anhydride (2 drops). The mixture was boiled and 1 drop of concentrated sulphuric acid added after cooling. A green colour indicated the presence of sterols. For tannins, 1 g of extract was dissolved in methanol and 2 drops of ferric chloride were added, and the mixture observed for appearance of an olive green colour (which is indicative of the presence of tannins) [8]. Alkaloids were tested by thin-layer chromatography (TLC) on aluminium plates coated with silica gel. Each extract (0.5 g) was dissolved in methylene chloride, spotted on the TLC plate, and resolved using a mobile phase constituted of 20 % hexane/ethylacetate. Resulting spots were developed by spraying the plate with Drangedoff’s reagent, and shiny yellow spots indicated the presence of alkaloids [25]. The presence of flavonoids was revealed by formation of crimson red colour when 0.1 g extract is dissolved in methanol with few fragments of magnesium ribbons and 1 drop of concentrated hydrochloric acid [25].

### Statistical analysis

To determine IC_50_ values of active extracts, the filaricidal activity data obtained were analysed using GraphPad Prism 6.0 (GraphPad Prism INC., CA, USA). The logarithm of the extract concentration was plotted against its activity determined by microscopy, to obtain a nonlinear regression curve –fitting and a variable slope sigmoidal dose- response curve.

## Results

A total of 14 extracts were prepared from different parts of the two plants using solvents of different polarities: hexane followed by methylene chloride and then methanol, sequentially. Generally, the percentage yields of hexane extracts were highest while those of methanol were lowest (Table 1).

**Table 1:** Plant parts and yield of crude extracts obtained with the different solvents

Plant name	Plant part (dry weight)	Parameter	Hexane (hex) extracts	Methylene chloride (mc) extracts	Methanol (met) extracts
*Tragia benthami*	Leaves (L) (131.05 g)	Extract code	TBLhex	TBLmc	TBLmet
		Yield (%)	4.20	1.31	0.08
	Roots (R) (83.21 g)	Extract code	TBRhex	TBRmc	TBRmet
		Yield (%)	0.29	0.24	0.60
*Piper umbellatum*	Leaves (L) (75.70 g)	Extract code	PULhex	PULmc	PULmet
		Yield (%)	2.97	1.78	0.61
	Roots (R) (75.70 g)	Extract code	PURhex	PURmc	PURmet
		Yield (%)	1.88	1.51	0.62
	Seeds (S) (33.54 g)	Extract code	PUShex	PUSmc	PUSmet
		Yield (%)	0.54	01.19	ND

*TBL* Tragia benthami leaves, *TBR* Tragia benthami roots, *PUL Piper umbellatum* leaves, *PUR Piper umbellatum* roots, *PUS Piper umbellatum* seeds, *hex* hexane, *mc* methylene chloride, *met* methanol, *ND* not done

**Fig. 1:**
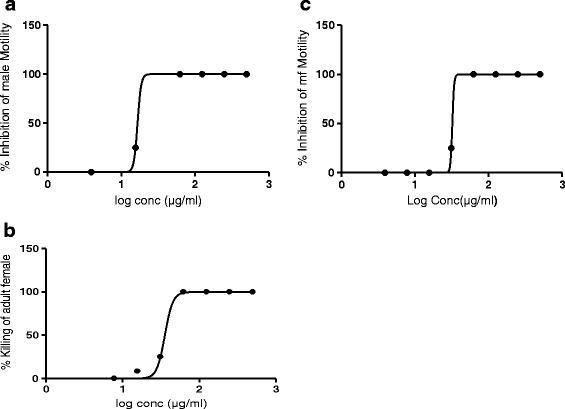
Dose dependent effect of (**a**) PULmc on adult *O. ochengi* male worms; (**b**) PULmc on adult *O. ochengi* female worms; (**c**) PULmc on *O. ochengi* microfilariae

In the primary screens on *O. ochengi*, at 500 μg/mL, 13 from the 14 extracts showed 100 % activity on adult male worms and mf while 9 had 100 % activity also on adult females (Table 2). Since activity on adult female worm is the most desirable property of an onchocerciasis drug, only these 9 extracts were selected for continuation of the study. They were screened at eight concentrations (500 μg/mL - 3.9 μg/mL) on the *O. ochengi* parasite stages, in experiments to determine IC_50_ values. The filaricidal activities of each extract showed a dose dependent response for both adult worms and mf (Figs. 1a, b and c). IC_50_ values generated using GraphPad prism version 6.0 (Graphpad software INC, USA) are shown in Tables 3 and 4. Of these 9 extracts, the methylene chloride preparation of *P. umbellatum* leaves (PULmc) was the most active on *O. ochengi* adult male and female worms, with IC_50_ values of 16.63 μg/mL and 35.65 μg/mL, respectively; and 125 μg/mL on the microfilariae (Table 3). For *T. Benthami,* only the hexane extract of the roots (TBRhex) showed 100 % activity on the adult female *O. ochengi* in the primary screens and did not show very encouraging results in experiments to determine IC_50_ values (Table 3).

**Table 2:** Mean percent activity of crude extracts on *Onchocerca ochengi* adult worms and mf in primary screens

Plant or control	Extract code	Concentration tested	Activity on *O. ochengi* male (%)	Activity on *O. ochengi* female (%)	Activity on *O. ochengi* mf (%)
*Tragia benthami*	TBLhex	500 μg/mL	100	91.25	75
	TBLmc	500 μg/mL	100	87.5	100
	TBLmet	500 μg/mL	100	56.25	100
	TBRhex	500 μg/mL	100	100	100
	TBRmc	500 μg/mL	100	93.75	100
	TBRmet	500 μg/mL	100	12.5	100
*Piper unbellatum*	PULhex	500 μg/mL	100	100	100
	PULmc	500 μg/mL	100	100	100
	PULmet	500 μg/mL	100	100	100
	PURhex	500 μg/mL	100	100	100
	PURmc	500 μg/mL	100	100	100
	PURmet	500 μg/mL	100	100	100
	PUShex	500 μg/mL	100	100	100
	PUSmc	500 μg/mL	100	100	100
Negative control	NA	2 % DMSO	0	0	0
Auranofin (Positive control)	NA	10 μM	100	100	100

*TBL* Tragia benthami leaves, *TBR* Tragia benthami roots, *PUL Piper umbellatum* leaves, *PUR Piper umbellatum* roots, *PUS Piper umbellatum* seeds, *hex* hexane, *mc* methylene chloride, *met* methanol, *NA* not applicable

**Table 3:** IC_50_ values, CC_50_s and Selectivity index (SI) values of the *O. ochengi* adult female hits on the different *O. ochengi* parasite stages tested

		Extract								
Nature of parameter	Parameter	TBRhex	PULhex	PULmc	PULmet	PURhex	PURmc	PURmet	PUShex	PUSmc
Inhibition of adult male worm motility	IC_50_ (μg/mL)	131.7	33.26	16.63	31.25	43.22	48.89	34.89	32.82	43.22
	CC_50_ (μg/mL)	375	93.75	93.75	93.73	46.88	93.75	93.75	46.88	46.88
	SI	2.8	2.8	5.6	3.0	1.1	1.9	2.7	1.4	1.1
Inhibition of formazan formation by female worms	IC_50_ (μg/mL)	213.5	67.87	35.65	125.30	37.36	162.70	125.30	37.79	37.36
	CC_50_ (μg/mL)	375	93.75	93.75	93.73	46.88	93.75	93.75	46.88	46.88
	SI	1.8	1.4	2.6	0.75	1.3	0.6	0.7	1.2	1.3
Inhibition of mf motility	IC_50_ (μg/mL)	154.8	62.50	125.0	31.25	55.61	62.50	31.25	31.25	125.0
	CC_50_ (μg/mL)	375	93.75	93.75	93.73	46.88	93.75	93.75	46.88	46.88
	SI	2.4	1.5	2.9	3.0	1.5	1.7	1.5	1.5	1.5

**Table 4:** Phytochemical analysis of extracts most active on *O. ochengi* female worms

Class of compound	PULmc	PURhex	PUShex
Saponins	+	+	−
Sterols	+++	+	+++
Tanins	+	+	−
Alkaloids	−	−	−
Flavonoids	+	+	+

+++: highly present +: Present; −: Absent.; The extracts codes are *PUL mc*, methylene chloride of *P. umbellatum* leaves, *PUR hex* hexane extracts of *P. umbellatum* roots, *PUS hex* hexane extract of *P. umbellatum* seeds

**Fig. 2:**
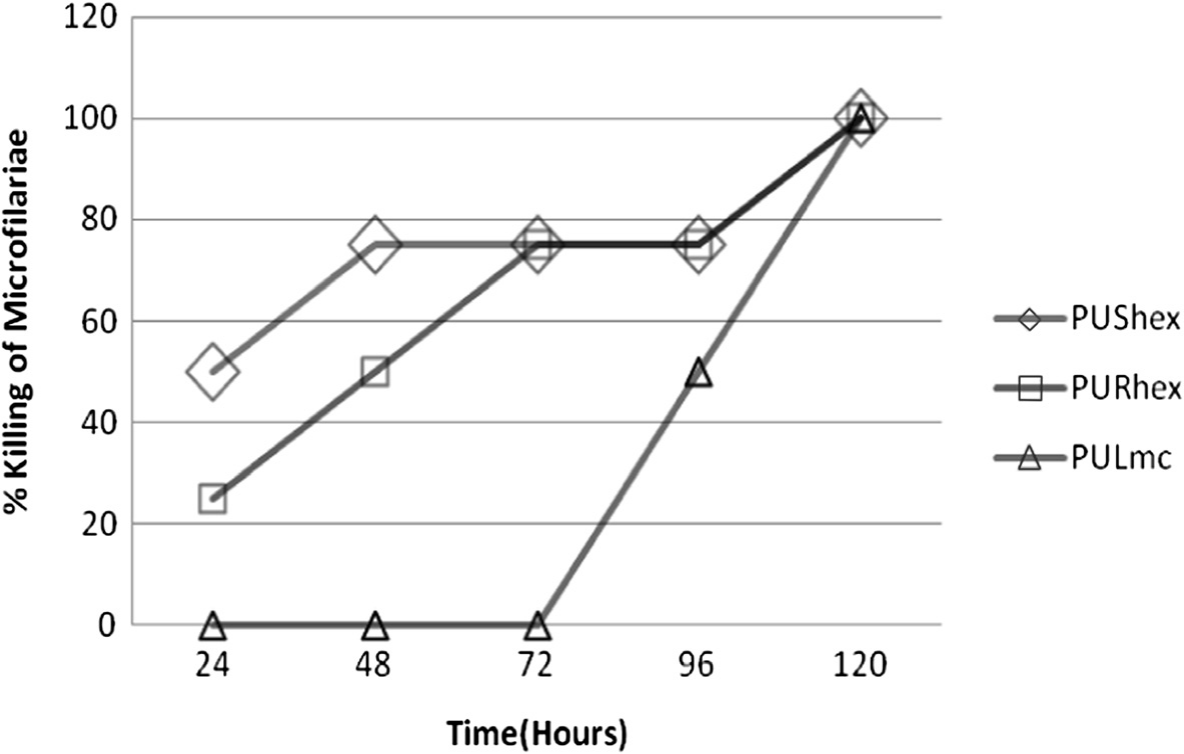
Time dependent killing of *O. ochengi* microfilariae by most active extracts at their IC_100_ concentrations

On the mf, the most active extracts were from *P. umbellatum* and included hexane extracts from seeds and roots (PUShex and PURhex, respectively, with IC_50_ of 31.25 μg/mL for both), as well as methylene chloride extract from leaves (PULmc IC_50_ of 32.23 μg/mL). Although PUShex and PURhex had the lowest and similar IC_50_ values on the *O. ochengi* mf (31.25 μg/mL for the 2 extracts), PUShex showed more rapid killing in time-dependent studies (Fig. 2).

The most active extracts on adult *O. ochengi* female worms were PULmc, PURhex and PUShex. These 3 extracts were further screened on *L. loa* mf at 8 concentrations (500–3.91 μg/mL) and the IC_50_ values obtained were 35.12, 16.12, and 13.90 μg/mL for PULmc, PURhex and PUShex respectively. All the three extracts were more active on *L. loa* mf than *O. ochengi* mf, with PUShex showing the lowest IC_50_ of 13.10 μg/mL on *L. loa* mf.

The cytotoxic effects of the interesting extracts were evaluated on monkey kidney epithelial cells (LLC-MK2) by microscopy alongside the mf screens. With the exception of PURmc and PURmet, all the extracts were more toxic to the *O. ochengi* female worms than to the cells, as reflected by their selectivity indices (SI) (Table 3). On *O. ochengi* mf, PULmc recorded the highest SI of 2.9 while PURhex, PURmet and PUShex recorded the lowest SI of 1.5 (Table 3). On adult males, PULmc recorded the highest SI of 5.6, while PURhex recorded the lowest SI of 1.1. Finally, on adult females, PULmc also recorded the highest SI of 2.6 while PURmc recorded the lowest SI of 0.6 (Table 3).

The 3 most active extracts on *O. ochengi* adult female and mf (PULmc, PURhex and PUShex) showed no sign of acute toxicity to Balb/c mice at a limit dose of 2000 mg/kg body weight when the extracts were given orally and the animals followed-up for 24 h, and 14 days post-treatment. The average weights of the mice increased from 18 g pre-treatment to 22.7 g post-treatment. When compared with the control group treated with vehicle (2 % DMSO dissolved in distilled water, and given at a dose of 0.2 mL/kg body weight), there was no significant difference in food and water intake. No change was observed in agility, physical appearance and behaviour of the mice 24 h post-treatment. Likewise, there was no loss of fur as well as no change in skin and mucous membrane. The test and control groups were indistinguishable from one another on the basis of their appearances and physical activity at the end of the study period (data not shown).

Phytochemical screening of the 3 most active extracts revealed the presence of saponins, steroids, tannins and flavonoids (Table 4).

## Discussion

The aim of the present study was to assess the activity of two medicinal plants, *T. benthami* and *P. umbellatum* on *O. ochengi* and *L. loa*. Extracts from the plants are locally used in the North West Region of Cameroon for the treatment of onchocerciasis. A total of 14 crude extracts of varying solubilities were prepared from parts of the plants and tested in previously standardised assays. Interestingly, 9 of the 14 extracts showed 100 % activity when screened in vitro at 500 μg/mL against *O. ochengi* adult female worms and mf and so were subjected to lower drug concentration screens, which also showed promising results with IC_50_ values reaching down to 16.63 μg/mL.

Previous studies have shown that some Cameroonian medicinal plants, including *Magaritaria discoidea*, *Homalium africanum*, *Craterispermum laurinum*, *Morinda lucida* and *Cyperus articulatus* also have anti *O. ochengi* activity [11–13]. The demonstration of such good activity by these two plants has come to expand the list. Additionally, two clear trends have emerged, the first being the high hit rate of such medicinal plant preparations on the *Onchocerca* parasites in vitro, and the second being that extracts from anti-*Onchocerca* medicinal plants also generally show activity against multiple pathogens. For example, extracts of *P. umbellatum* have shown activity against many coliform bacteria [19], and has also been used to treat certain physiological or biochemical disorders [17].

Selection of the 9 extracts from the primary screen data for further studies was mainly based on their good activities on adult female *O. ochengi* worms. Activity on female worm is most important because this is the stage that produces the microfilariae that generate pathologies and because from this and previous studies, it is the most difficult stage to kill with drugs. In these secondary screens, the extracts showed a dose and time dependent activity on parasites and the highly active ones on *O. ochengi* were PULmc (lowest IC_50_ of 16.63 μg/mL on adult males), PURhex (lowest IC_50_ of 37.36 μg/mL on adult females) and PUShex (lowest IC_50_ of 33.26 μg/mL on adult males) (Table 3). These three extracts were also very active on *L. loa* mf. PURhex and PUShex exhibited higher activity on *L. loa* mf than on *O. ochengi* mf (IC_50_ of 16.12 μg/mL and 13.90 μg/mL versus 31.25 μg/mL each on *O. ochengi* mf, respectively). This suggests that these two extracts cannot be safely used as phytomedicine to treat onchocerciasis in individuals with high *L. loa* mf load, as this may lead to the severe adverse reaction previously reported [5]. However, PULmc exhibited slightly less, but similar activity on *L. loa* and *O. ochengi* mf (IC_50_ values of 35.12 μg/mL versus 32.13 μg/mL, respectively). Further fractionation or isolation of compounds from the extracts is recommended since this may result in some degree of resolution of anti-*Onchocerca* and anti-*L. loa* components, leading to the elimination of compounds that selectively kill the *L. loa* mf.

After investigating the filaricidal activity of the extracts, it was necessary to investigate their safety. This was done by evaluating cytotoxicity on LLC-MK2 cells for the 9 extracts that were active on female worms, and acute toxicity in Balb/c mice for the 3 most active extracts, which were part of the 9 female hits. With the exception of PURmc and PURmet, all the extracts tested were more toxic to the *O. ochengi* worm stages than to cells, as reflected by their SIs (Table 3). The safest extract, PULmc, recorded the highest selectivity indices of 5.6, 2.6 and 2.9 for *O. ochengi* adult male, adult female and mf, respectively. A starting point of 5.6 is good enough to encourage further refinement of an extract or development of an active principle contained there in. When tested for toxicity in vivo, all 3 most active extracts (PULmc, PURhex and PUShex) showed no sign of acute toxicity to Balb/c mice. This suggests that these 3 extracts could be safely used in phytomedicines for onchocerciasis treatment in areas where *L. loa* is not prevalent, and justifies their popular use in traditional medicine in the North West Region of Cameroon as anti-*Onchocerca* remedies. Worth noting is the fact that toxic and mutagenic concentrations of different substances could differ greatly between cells from human and other species, indicating that results of such studies cannot always be extrapolated from animal to human situations [26].

Pharmacological potential of plants is attributed to presence of wide array of phytochemical compounds in them [27, 28]. Therefore, in current study an effort was made to explore potential active ingredients from the studied extracts. The phytochemical screening of the three promising extracts revealed the presence of steroids, flavanoids, and tannins/saponins (Table 4), suggesting that active principles in the extracts may be from the aforementioned groups of compounds. This corroborates previous findings in which microfilaricidal extracts (activity against *O. ochengi* mfs) from *Margaritaria discoidea* and *Homalium africanum* contained sterols and terpenoids [11]. An extract from *Annona senegalensis*, which inhibited survival of *O. ochengi* adult worms, was reported to be rich in saponins, tannins and flavonoids [29]. Also, the presence of sterols, saponins, and/or flavonoids was reported in the fractions from *Craterispermum laurinum* and *Morinda lucida* which were active on *O. ochengi* adult worms in vitro [13]. Likewise, crude extracts of *Artemisia annua* were shown to possess a plethora of other compounds including flavonoids, which enhance the in vitro activity of artemisinin against the parasite *Plasmodium falciparum* [10].

## Conclusion

In order to assess the anti-filarial properties of *Piper umbellatum* L. and *Tragia benthami* B., a total of 14 extracts were prepared from the plants and screened in vitro against *Onchocerca ochengi* parasite and *L. loa* microfilariae. Cytotoxicity and acute toxicity of active extracts were tested on monkey kidney cells and Balb/c mice, respectively. The three most active extracts on *Onchocerca ochengi* females were also highly active on *Loa loa* microfilariae, with IC_50_s of 35.12 - 13.9 μg/mL. Overall, results from this study suggests that crude extracts of different polarities of *P. umbellatum* and *T. benthami* exhibit selective filaricidal activity and could serve as potential sources of new drugs against onchocerciasis. It also validates the traditional use of these plants in the treatment of onchocerciasis.

## Abbreviations

CCM: Complete culture medium
DMSO: Dimethyl sulfoxide
ICM: Incomplete culture medium
*L. loa*: *Loa loa*
Mf: Microfilariae
*O. ochengi*: *Onchocerca ochengi*
*O. volvulus*: *Onchocerca volvulus*
PULhex: Hexane extract of *Piper unbellatum* leaves
PULmc: Methylene chloride extract of *Piper unbellatum* leaves
PULmet: Methanol extract of *Piper unbellatum* leaves
PURhex: Hexane extract of *Piper unbellatum* roots
PURmc: Methylene chloride extract of *Piper unbellatum* roots
PURmet: Methanol extract of *Piper unbellatum* roots
PUShex: Hexane extract of *Piper unbellatum* seeds
PUSmc: Methylene chloride extract of *Piper unbellatum* seeds
SI: Selectivity index
TBLhex: Hexane extract of *Tragia benthami* leaves
TBLmc: Methylene chloride extract of *Tragia benthami* leaves
TBLmet: Methanol extract of *Tragia benthami* leaves
TBRhex: Methanol extract of *Tragia benthami* roots
TBRmc: Methylene chloride extract of *Tragia benthami* roots
TBRmet: Methanol extract of *Tragia benthami* roots
TLC: Thin-layer chromatography

## References

[1] World Health Organization: Global Initiative for the Elimination of Avoidable Blindness, Action Plan 2006 – 2011. WHO; 2007:29 [http://www.who.int/blindness/Vision2020_report.pdf]

[2] Brattig NW (2004). Pathogenesis and host responses in human Onchocerciasis: impact of Onchocerca filariae and *wolbachia* endobacteria. Microbes Infect.

[3] Wanji S. Rapid assessment procedures for loiasis. Report of a multi-centre study. UNDP/World Bank/ WHO special programme for research and training in tropical diseases (TDR). 2001; 38p.

[4] Thomsom MC, Obsomer V, Kamgno J, Gardon J, Wanji S, Takougang I, Enyong P, Remme JH, Molyneex DH, Bossinesg M (2004). Mapping the distribution *of Loa loa* in Cameroon in support of the African programme for Onchocerciasis control. Filaria J.

[5] Turner JD, Tendonfor N, Esum M, Johnson KL, Langley RS, Ford L, Faragher B, Specht S, Mand S, Hoeraf A, Enyong P, Wanji S, Taylor MJ (2010). Macrofilaricidal activity after doxycycline onlytreatment of *Onchocerca volvulus* in an area of *Loa loa* co-endemicity: a randomized control trial. PLoS Negl Trop Dis.

[6] Cupp EW, Mackenzie CD, Unnasch TR (2011). Importance of Ivermectin to human onchocerciasis: past, present, and the future. Res Rep Trop Med.

[7] WHO (The World Health Organization): Traditional medicine. WHO fact sheet N°134.

[8] Kokate CK, Purohit AP, Gokhale SB: Practical Pharmacognosy .2^nd^ edition, Nirali Prakashan, Pune. 1994; 54–60.

[9] Lindley D (1987). Merck’s new drug free to WHO for river blindness programme. Nature.

[10] Phillipson JD (2000). Phytochemistry and medicinal plants. Phytochemistry.

[11] Cho-Ngwa F, Abongwa M, Ngemenya MN, Nyongbela KD (2010). Selective activity of extracts of *margaritaria discoidea* and *homalium Africanum* on *Onchocerca ochengi*. BMC Complement Altern Med.

[12] Metuge JA, Nyongbela KD, Mbah J, Samje M, Fotso G, Babiaka SB, Cho-Ngwa F (2014). Anti- Onchocerca activity and phytochemical analysis of an essential oil from *Cyperus articulatus L*. BMC Complement Altern Med.

[13] Samje M, Metuge J, Mbah J, Nguesson B, Cho-Ngwa F (2014). In vitro anti- Onchocerca ochengi activities of extracts and chromatographic fractions of *Craterispermum laurinum* and *Morinda lucida*. BMC Complement Altern Med.

[14] Trees AJ, Graham SP, Renz A, Bianco AE, Tanya V (2000). *Onchocerca ochengi* infections in cattle as a model for human Onchocerciasis: recent developments. Parasitol.

[15] Panda D, Dash SK, Dash GK (2012). Phytochemical examination and antimicrobial activity of various solvent extracts and the selected isolated compounds from roots of *tragia involucrata* Linn. Int J Pharm Sci Drug Res.

[16] Kalaivanan M, Jesudass LL (2012). Pharmacological studies on ethanol extract of *Tragia Plukenetii* R. Smith IOSR J Pharm.

[17] Nwafor PA, EmemEkpo E, Udofia EE, Smith ME (2012). Effects of methanol extracts of *Piper umbellatum* leaves on contraceptive and sexual behavior in rodents. Niger J Pharm Appl Sci Res.

[18] Ejele AE, Duru IA, Oze RN, Iwu IC, Ogukwe CE (2012). Comparison of antimicrobial potential of *Piperumbellatum, Piper guineense, Ocimum gratissimum* and *Newbouldia laevis* extracts. Int Res J Biochem Bioinfo.

[19] Nwauzoma AB, Dawari SL (2013). Studies on the phytochemical properties and proximate analysis of *Piper umbellatum* Linn from Nigeria. Am J Res Com.

[20] Cho-Ngwa F, Daggfeldt A, Titanji VPK, Gronvik K (2005). Preparation and characterisation of specific monoclonal antibodies for the detection of adult worm infections in onchocerciasis. Hybridoma.

[21] Bulman CA, Bidlow CM, Lustigman S, Cho-Ngwa F, Williams D, Rascón AA, Tricoche N, Samje M, Bell A, Suzuki B, Lim KC, Supakorndej N, Supakorndej P, Wolfe AR, Knudsen GM, Chen S, Wilson C, Ang KH, Arkin M, Gut J, Franklin C, Marcellino C, McKerrow JH, Debnath A, Sakanari JA (2015). Repurposing auranofin as a lead candidate for treatment of lymphatic filariasis and onchocerciasis. PLoS Negl Trop Dis.

[22] Goussard B, Garin Y, Ivanoff B (1985). Loa loa: a simple method for isolation of microfilariae from blood with production of antigen in extraction medium. Trans R Soc Trop Med Hyg..

[23] Organisation for Economic Co − operation and Development (OECD*)*. Guidelines for testing chemicals. Paris Mongragh. 2001.

[24] Paech K, Tracey MV: Modern methods of Plant analysis. 3^rd^ edition. Springer-Verlag Berlin Heidelberg. 1 995; 467–478 .

[25] Pulok K, Mukherjee. Quality control of Herbal Drugs: an approach to evaluation of botanicals. New Delhi: Business Horizons; 2002. p. 540-542.

[26] Ashraf A, Sarfraz RA, Mahmood A, Din MU (2015). Chemical composition and *in vitro* antioxidant and antitumor activities of *Eucalyptus camaldulensis* Dehn. leaves. Indust CropProd.

[27] Ashraf A, Sarfraz RA, Rashid MA, Shahid M (2014). Antioxidant, antimicrobial, antitumor, and cytotoxic activities of an important medicinal plant (*Euphorbia royleana*) from Pakistan. J Food Drug Ana.

[28] Singh B, Gupta RS (1985). Species-specific differences in the toxicity and Mutagenicity of the AnticancerDrugs mithramycin, chromomycin A3, and olivomycin. Cancer Res.

[29] Ndjonka D, Agyare C, Lüersen K, Djafsia B, Achukwi D, Nukenine EN, Hensel A, Liebau E (2011). *In vitro* activity of Cameroonian and Ghanaian medicinal plants on parasitic (Onchocerca ochengi) and free-living (Caenorhabditis elegans) nematodes. J Helminthol.

